# Segregation Predicts COVID-19 Fatalities in Less Densely Populated Counties

**DOI:** 10.7759/cureus.21319

**Published:** 2022-01-17

**Authors:** Becky Li, Ryan J Quinn, Salimah Meghani, Jesse L Chittams, Vijay Rajput

**Affiliations:** 1 School of Medicine, Nova Southeastern University Dr. Kiran C. Patel College of Allopathic Medicine, Fort Lauderdale, USA; 2 Biostatistics, University of Pennsylvania School of Nursing, Philadelphia, USA; 3 Biobehavioral Health Sciences, University of Pennsylvania School of Nursing, Philadelphia, USA; 4 Medical Education, Nova Southeastern University Dr. Kiran C. Patel College of Allopathic Medicine, Fort Lauderdale, USA

**Keywords:** barriers to healthcare, residential segregation, covid-19, health disparities, social determinants of health

## Abstract

Aim

It is well known that social determinants of health (SDoH) have affected COVID-19 outcomes, but these determinants are broad and complex. Identifying essential determinants is a prerequisite to address widening health disparities during the evolving COVID-19 pandemic.

Methods

County-specific COVID-19 fatality data from California, Illinois, and New York, three US states with the highest county-cevel COVID-19 fatalities as of June 15, 2020, were analyzed. Twenty-three county-level SDoH, collected from County Health Rankings & Roadmaps (CHRR), were considered. A median split on the population-adjusted COVID-19 fatality rate created an indicator for high or low fatality. The decision tree method, which employs machine learning techniques, analyzed and visualized associations between SDoH and high COVID-19 fatality rate at the county level.

Results

Of the 23 county-level SDoH considered, population density, residential segregation (between white and non-white populations), and preventable hospitalization rates were key predictors of COVID-19 fatalities. Segregation was an important predictor of COVID-19 fatalities in counties of low population density. The model area under the curve (AUC) was 0.79, with a sensitivity of 74% and specificity of 76%.

Conclusion

Our findings, using a novel analytical lens, suggest that COVID-19 fatality is high in areas of high population density. While population density correlates to COVID-19 fatality, our study also finds that segregation predicts COVID-19 fatality in less densely populated counties. These findings have implications for COVID-19 resource planning and require appropriate attention.

## Introduction

The COVID-19 pandemic is predicted to widen the health gap for minorities with pre-existing inequalities, such as Black, Hispanic, and Native American populations [1,2]. Minorities and low-income populations are vulnerable due to inadequate healthcare access, fewer opportunities to clarify misinformation (due to reduced access to high-quality information channels), and susceptibility to comorbidities [1,3].

The disproportionate consequences of COVID-19 are exacerbated by the inability of minorities and low-income families to maintain adequate social distancing, as they constitute a great portion of the frontline and essential workforce and reside in densely populated homes [1]. Frontline occupations include nurses, delivery workers, and others who could not work from home. In the United States (US), 41.2% of frontline workers identify as a person of color, and more than a third of the frontline workers are supporting low-income families [4]. We, therefore, investigated social determinants of health (SDoH) as they are associated with disparities in COVID-19 transmission and outcomes [5].

Notably, the current literature investigating SDoH and COVID-19 together at the county level is limited, particularly in methods of analysis. Previous studies have identified county-level risk factors affecting COVID-19 susceptibility and mortality utilizing bivariate or regression analysis [6-10]. Only one study has used the tree-based machine-learning analytical method but focused on county-level COVID-19 incidence in a single state [11]. Our research is centered on contextualizing county-level data in COVID-19 outcomes rather than susceptibilities. Employing 23 county-level SDoH and a unique tree-based analytical lens, our study aimed to identify the key county-level social determinants of COVID-19 fatality in the midst of the first COVID wave in three US states: New York, Illinois, and California.

## Materials and methods

Study population and methods

We selected three US states, namely California, Illinois, and New York, containing counties with the highest absolute COVID-19 fatalities as of June 15, 2020 [6]. Total cumulative COVID-19 cases and fatalities were gathered from state department of health websites in June 2020 [12-14]. County attributes were simultaneously collected from County Health Rankings & Roadmaps (CHRR) which assembled widely used county-level data from publicly available datasets [15-18]. Population density was measured in persons per square mile [19,20]. Residential segregation was studied as an index of dissimilarity between white and non-white populations on a scale of 0 (integration) to 100 (segregation) [21]. Preventable hospital stays were defined as the rate of hospital stays for conditions that can be treated as outpatient, per 100,000 Medicare enrollees. This study was granted exemption by the Nova Southeastern University Institutional Review Board (IRB) for not involving human subjects per the federal regulations (IRB #2020-229). Data analysis was conducted in August and September 2020.

Statistical analysis

Twenty-three county-level predictors of interest were analyzed through summary statistics. The outcome measure was COVID-19 fatality rate calculated as deaths per 100,000 county population. Inclusion criteria was implemented to only include counties with a population density between 1.5 to 50,000 persons per square mile (n=198). Bivariate associations between SDoH and continuous COVID-19 fatality rate were assessed with Spearman correlations. A median split performed on the population-adjusted COVID-19 fatality rate created a high/low fatality indicator used in subsequent predictive modeling using decision tree analysis.

The decision tree was generated using the HPSPLIT procedure in SAS (Statistical Analysis System; SAS Institute, Cary, USA) to visualize associations among SDoH and to explore the profile of counties most at risk for high COVID-19 fatality. We selected decision tree analysis for its robustness, ease of interpretation, and simplification of complex relationships seen in SDoH [22]. Tree analyses are built from known data and subsequently utilized to predict future outcomes [23]. In certain analyses, decision trees have been superior to logistic regression in predicting case outcomes, particularly outcomes that behave in a non-linear fashion [24].

The decision tree method employs machine learning techniques to determine a parsimonious model, defining profiles which best classify counties by outcome status [22]. Tree building starts at the root node, which contains all the data (county fatalities), and is partitioned recursively into child nodes until it reaches its terminal nodes. The split is based on selecting the predictor that best discriminates between high and low fatality counties [23]. Ten-fold cross-validation was employed for pruning and validation of the final tree [25]. Model accuracy was evaluated based on sensitivity, specificity, and area under the receiver operating characteristic curve (AUC). Sensitivity analyses confirmed variable selection results. In a follow-up analysis, Kruskal-Wallis tests were applied to analyze how counties in certain model-defined profiles differed from other counties based on predictors of interest.

## Results

**Figure 1:**
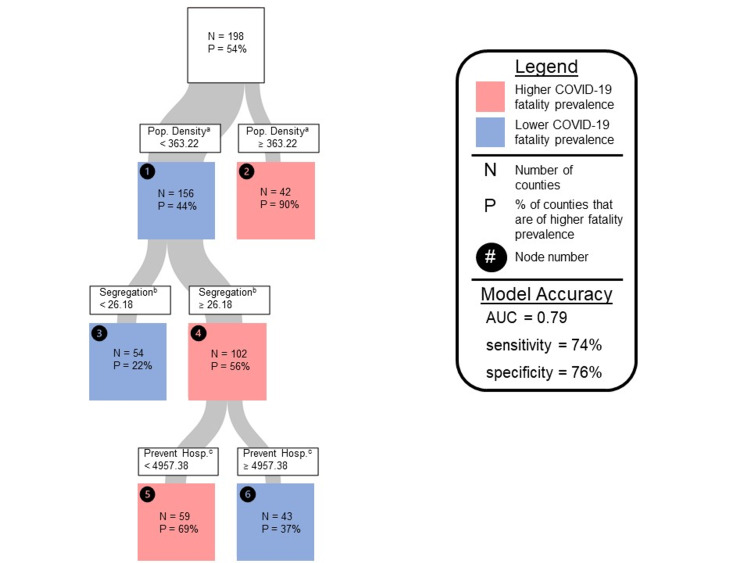
Decision tree analysis of social determinants of health and COVID-19 fatalities Pop. - population; Hosp. - hospitalizations; N - number of counties; P - % of counties that are of higher fatality prevalence; AUC - area under the receiver operating characteristic curve ^a^Population density in persons per square mile. Data retrieved on June 15, 2020 from US Census Bureau [19,20]. ^b^Residential segregation as an index of dissimilarity (between white vs. non-white). Data retrieved on June 15, 2020 from County Health Rankings [16-18]. ^c^Preventable hospital stays as hospitalization rate of outpatient-sensitive conditions per 100,000 Medicare enrollees. Data retrieved June on 15, 2020 from County Health Rankings [16-18].

Table 1 presents summary statistics on variables of interest. The median fatality rate was 4.5 deaths per 100,000 county population. Spearman correlations identified seven county-level SDoH exhibiting a moderate association (r>0.3) with the continuous COVID-19 fatality rate (Table 2). Upon further analysis, the decision tree model identified population density, residential segregation, and preventable hospitalizations to be key predictors of counties with high COVID-19 fatality rates (Figure 1). The tree had four total terminal nodes reflecting county profiles with a color characterization of low COVID fatality (blue) or high COVID fatality (pink). The model area under the curve (AUC) was 0.79, with 74% sensitivity, 76% specificity, and 25% misclassification rate. The average sensitivity, specificity, and misclassification rates among cross-validation subsamples were 64%, 54%, and 40%, respectively.

**Table 1:** Descriptive statistics on county attributes and COVID-19 outcomes CA - California; IL - Illinois; NY =- New York; IQR - interquartile range; PCP - primary care physicians ^a^Data was retrieved on June 15, 2020 from the California, Illinois, and New York Department of Health [12-14] ^b^Data was retrieved on June 15, 2020 from US Census Bureau [19,20] ^c^Data was retrieved on June 15, 2020 from County Health Rankings & Roadmaps [16-18]

Measure	Overall median (IQR)	CA median (IQR)	IL median (IQR)	NY median (IQR)
COVID-19-related outcomes				
COVID-positive cases^a^ (# total positive cases as defined by each state department of health)	131 (854)	254 (1941.5)	45 (211)	253.5 (1766.5)
COVID-19 fatalities^a ^(# fatalities/100,000 county population)	4.5 (33)	4 (43)	1.5 (15)	21 (90.5)
Sociodemographic factors				
Population density^b^ (# persons per square mile estimated from 2019 population and 2010 land area)	87.9 (200.17)	111.81 (380.88)	56.64 (86.8)	120.91 (376.86)
Elderly^c^ (% adults ages 65 and older)	18.8 (4.5)	15.8 (7.45)	19.3 (2.9)	18.45 (2.55)
Black (% non-Hispanic Black or African American)	2.55 (5.8)	2 (3)	3.55 (7.1)	4 (7.1)
Non-Hispanic White^c^ (%)	82.9 (26.9)	48.55 (37.55)	90 (12.8)	86.7 (17.9)
Female^c^ (%)	50.3 (1.1)	50.2 (1.3)	50.35 (1)	50.4 (1.3)
Rural^c^ (%)	40.85 (47)	15.15 (35.75)	44.4 (36.3)	46.75 (41.65)
High school graduation rate^c^ (% ninth-graders who graduate in 4 years)	86 (7)	85 (6)	88.5 (8)	85 (4.5)
Some college^c^ (% adults ages 25-44 with some post-secondary education)	62.5 (11)	60 (17)	63 (8)	62 (9.5)
Unemployment rate^c^ (% people age ≥ 16 unemployed but seeking work)	4.6 (1.3)	4.65 (2.8)	4.8 (0.9)	4.35 (1)
Income inequality^c^ (ratio of 80th percentile income to 20th percentile income)	4.5 (0.6)	4.7 (0.5)	4.35 (0.7)	4.5 (0.6)
Residential segregation^c^ (index of dissimilarity ranging 0-100, used in the American Community Survey where higher values indicate greater residential segregation between non-White and White county residents)	33 (18)	25 (10.5)	36.5 (16)	39 (15.5)
Food insecurity^c^ (%)	11 (3)	12.5 (3.5)	11 (3)	11 (2)
Limited access to healthy foods^c^ (% low-income population who do not live close to grocery store)	5 (4)	5 (5)	5.5 (4)	4 (3)
Physical environment				
Severe housing problems^c^ (% households at least 1 of 4 housing problems: overcrowding, high housing costs, lack of kitchen facilities, or lack of plumbing facilities)	15 (10)	23 (4)	11 (4)	15 (4)
Homeowner percentage^c^ (% occupied housing units that are owned)	71 (12)	61 (9)	75 (7)	71 (6.5)
Severe housing cost burden^c^ (% households that spend ≥ 50% household income on housing)	13 (7)	18 (3)	10 (4)	14 (4)
Drive alone to work^c^ (%)	80.5 (7)	76 (7.5)	83 (4)	80 (6)
Average traffic^c^ (traffic volume per meter)	152 (258)	215 (439.5)	89 (100)	296 (594.5)
Health behaviors				
Physical inactivity^c^ (% adults aged ≥ 20 who report no leisure-time physical activity)	25 (6)	21 (9)	26 (6)	26 (5)
Clinical care				
Uninsured^c^ (% persons under age 65 without health insurance)	6 (2)	8 (3)	6 (1)	5 (1)
Primary care physicians (ratio of county population to PCP)	1933 (1376)	1433 (1091.5)	2271.5 (1320)	1816 (1329)
Preventable hospital stays^c^ (rate of hospital stays for ambulatory-care sensitive conditions per 100,000 Medicare enrollees in a year)	4440.5 (1649)	3307.5 (1077)	5011.5 (1821)	4477.5 (1098)
Flu vaccinations^c^ (% fee-for-service [FFS] Medicare enrollees that had an annual flu vaccination)	46 (10)	41 (8.5)	44 (9)	50.5 (4.5)

**Table 2:** Strength of association between social determinants of health and COVID-19 mortality PCP - primary care physicians ^a^R-values as reported from Spearman’s Correlation test ^b^Data was retrieved on June 15, 2020 from US Census Bureau [19,20] ^c^Data was retrieved on June 15, 2020 from County Health Rankings & Roadmaps [16-18]

Social determinant	R value^a^
Population density^b^ (# persons per square mile)	0.54486
Average traffic^c^ (traffic volume per meter)	0.46935
Black (% non-Hispanic Black or African American)	0.44544
Residential segregation^c^ (index of dissimilarity ranging 0-100, used in the American Community Survey where higher values indicate greater residential segregation between non-White and White county residents)	0.43384
Flu vaccinations^c^ (% fee-for-service [FFS] Medicare enrollees that had an annual flu vaccination)	0.42261
Rural^c^ (% population)	-0.36288
Elderly^c^ (% adults ages 65 and older)	-0.34550
Food insecurity^c^ (%)	-0.26115
Primary care physicians^c^ (ratio of county population to PCP)	-0.24212
Unemployment rate^c^ (% people age ≥ 16 unemployed but seeking work)	-0.23788
Severe housing cost burden^c^ (% households that spend ≥ 50% household income on housing)	0.23565
Female^c^ (%)	0.22523
Non-Hispanic White^c^ (%)	-0.22470
Some college^c^ (% adults ages 25-44 with some post-secondary education)	0.21744
Severe housing problems^c^ (% households at least 1 of 4 housing problems: overcrowding, high housing costs, lack of kitchen facilities, or lack of plumbing facilities)	0.18491
Income inequality^c^ (ratio of 80th percentile income to 20th percentile income)	0.18212
Limited access to healthy foods^c^ (% low-income population who do not live close to grocery store)	-0.12510
Uninsured^c^ (% persons under age 65 without health insurance)	0.09888
Preventable hospital stays^c^ (rate of hospital stays for ambulatory-care sensitive conditions per 100,000 Medicare enrollees in a year)	0.09763
Homeowner percentage^c^ (% occupied housing units that are owned)	-0.09586
Drive alone to work^b^ (%)	-0.08880
Physical inactivity^c^ (% adults aged ≥ 20 who report no leisure-time physical activity)	-0.06643
High school graduation rate^c^ (%)	0.03359

Counties with population density ≥363.220 persons per square mile (p/mile^2^) were classified as high fatality. Of note, among counties with population density <363.220 p/mile^2^, those with residential segregation <26.180 were classified as low fatality. Interestingly, among counties with population density <363.220 p/mile^2^ and segregation ≥26.180, those with a lower preventable hospitalization rate per 100,000 Medicare enrollees (<4957.38) were classified as high COVID-19 fatality rate, as seen in terminal node number five (Figure 1). In a follow-up analysis, counties in node five exhibited significantly higher residential segregation, higher elderly percentage, lower severe housing issue percentage, and lower preventable hospitalization rate compared to other counties analyzed (p<0.05; see Table 3). Counties in node six, with the profile of population density <363,220 p/mile^2^, residential segregation ≥26.180, and preventable hospitalizations ≥4957.380 hospitalizations per 100,000 Medicare enrollees were predicted to have lower fatality rates (Figure 1).

**Table 3:** Descriptive statistics and Kruskal-Wallis analyses of Node 5 versus Node 6 and other counties excl. - excluding; PCP - primary care physicians ^a^Data was retrieved on June 15, 2020 from US Census Bureau [19,26] ^b^Data was retrieved on June 15, 2020 from County Health Rankings & Roadmaps [16-18] *p<0.05

Measure	Node 5 median	Other counties (excl. Node 5) median	Node 6 median	Node 5 vs. other counties p-value	Node 5 vs. Node 6 p-value
Sociodemographic factors					
Population density^a^ (# persons per square mile estimated from 2019 population and 2010 land area)	79.23	94.08	58.96	0.2884	0.0775
Elderly^b^ (% adults ages 65 and older)	19.1	18	19	0.0413*	0.7194
Black (% non-Hispanic Black or African American)	2.5	2.7	2.8	0.6341	0.9379
Non-Hispanic White^b^ (%)	86.6	79.6	90.4	0.0514	0.0148*
Female^b^ (%)	50.3	50.3	50.2	0.7788	0.5082
Rural^b^ (%)	43.3	38.9	58	0.1268	0.0339*
High school graduation rate^b^ (% ninth graders who graduate in 4 years)	86	87	89	0.8930	0.0820
Some college^b^ (% adults ages 25-44 with some post-secondary education)	63	62	60	0.3963	0.0130*
Unemployment rate^b^ (% people age ≥ 16 unemployed but seeking work)	4.5	4.7	4.9	0.4729	0.0091*
Income inequality^b^ (ratio of 80th percentile income to 20th percentile income)	4.5	4.5	4.4	0.6285	0.2752
Residential segregation^b^ (index of dissimilarity ranging 0-100, used in the American Community Survey where higher values indicate greater residential segregation between non-White and White county residents)	34	31	38	0.0087*	0.0180*
Food insecurity^b^ (%)	11	12	12	0.0964	0.0116*
Limited access to healthy foods^b^ (% low-income population who do not live close to grocery store)	5	5	5	0.2851	1.0000
Physical environment					
Severe housing problems^b^ (% households at least 1 of 4 housing problems: overcrowding, high housing costs, lack of kitchen facilities, or lack of plumbing facilities)	14	15	12	0.0279*	0.0503
Homeowner percentage^b^ (% occupied housing units that are owned)	72	71	73	0.5901	0.0703
Severe housing cost burden^b^ (% households that spend ≥ 50% household income on housing)	12	13	11	0.0973	0.0310*
Drive alone to work^b^ (%)	81	80	83	0.1043	0.0209*
Average traffic^b^ (traffic volume per meter)	152	152	99	0.5204	0.0758
Health behaviors					
Physical inactivity^b^ (% adults aged ≥ 20 who report no leisure-time physical activity)	25	25	28	0.7546	0.0005*
Clinical care					
Uninsured^b^ (% persons under age 65 without health insurance)	6	6	6	0.5261	0.4584
Primary care physicians^b^ (ratio of county population to PCP)	1931	1935	2438	0.8293	0.0342*
Preventable hospital stays^b^ (rate of hospital stays for ambulatory-care sensitive conditions per 100,000 Medicare enrollees in a year)	4163	4711	5795	0.0006*	< .0001>
Flu vaccinations^b^ (% fee-for-service [FFS] Medicare enrollees that had an annual flu vaccination)	46	45	45	0.1776	0.2218

## Discussion

This study sought to use the innovative decision tree model approach to identify relevant SDoH affecting COVID-19 fatalities in the US. While earlier studies have identified SDoH to be influential in COVID-19 related mortality, the key finding of our study is that even in counties of low population density, higher levels of segregation are substantially associated with high county-level COVID-19 related deaths.

Consistent with the Spearman correlations tested, population density and residential segregation remained important in the decision tree modeling. The population density was identified to be the most important county-level predictor of COVID-19 fatalities. This can be explained by denser areas having greater transmission rates, augmenting fatal outcomes. Despite population density being the most important factor, counties of low population density still exhibited relatively high COVID-19 fatality if there was a high degree of residential segregation. Residential segregation was measured in this study by the dissimilarity index, the most widely used measure of evenness comparing spatial distributions of different groups [26]. Measured as an index (0-100), it describes the percentage of white or non-white populations that would have to move to match the population distribution of the metropolitan (larger) area [21]. This study's data-driven decision tree approach determined the segregation index value of 26.180 to be the optimal threshold to be operationalized for predicting high county-level COVID-19 mortality rates. To our knowledge, there is no national standard that would otherwise define the parameters for "high" segregation. 

Residential segregation was the second most important county-level predictor, introducing the second split in our decision tree. Although legally banned since 1968, racial residential segregation has intact structures that continue to cause health disparities today [27]. Segregation affects other SDoH, such as socioeconomic (SES) status and poverty, resulting in poorer health outcomes for minorities. Segregation impairs SES status through diminishing access and resources for high-quality education and concentrating higher-pay jobs in areas outside of minority communities [27]. By creating areas of concentrated poverty, segregation has exposed disenfranchised populations' health to harms such as pollution, poor-quality infrastructure, and psychosocial stressors [28]. These harms result in disparities in income, life expectancy, and other SDoH that contribute to poor health outcomes [29]. Therefore, because elements of segregation are pervasive in driving social determinants of health, it is important to study the area of residence when investigating the mechanism of disease onset and progression. Previous studies have shown a positive association between segregation and county-level COVID-19 infection rates [8,10,30]. Our results show that segregation also predicts high COVID-19 fatality rates at the county level. Thus, it is essential to address segregation at the local level in addition to state and national interventions.

The potential relationship between population density and segregation may explain segregation's influence on COVID-19 outcomes in specifically less densely populated counties. In urban areas, population density has an inverse relationship with segregation. Anti-density zoning has restricted private property rights and kept population density low in targeted neighborhoods, increasing residential segregation [31]. Segregation conversely declines with urban population growth, diminishing segregation's influence on health outcomes in populated areas [32]. The observed negative relationship between population density and segregation in urban areas calls forth investigation of possible similar patterns in less dense areas.

Preventable hospital stays are defined as the rate of hospital stays for conditions that can be treated as outpatient per 100,000 Medicare enrollees. It is an indicator of unsatisfactory outpatient care or a pattern of excessively seeking urgent/emergency care [33]. Interestingly, our findings suggest that lower preventable hospital stays can predict COVID-19 fatalities in less dense but highly segregated counties. This finding is counterintuitive as it would mean that counties with higher COVID-19 fatalities are less likely to have Medicare enrollees excessively using urgent/emergency care for less urgent issues. This finding can potentially be explained by racial disparities, as this group of counties contained a significantly higher proportion of minorities who experience disproportionate barriers to accessing healthcare. Minorities have disproportionately faced higher COVID-19 mortality, which is related to their heightened exposure risk, illness severity, and barriers to testing [34]. Further analysis also showed that this group had a significantly greater housing cost burden, which also is recognized as a barrier to healthcare access [35, 36]. Further investigation is needed into other factors related to preventable hospital stays that can possibly explain this relationship.

Limitations

The study had the following limitations. First, we used data from three states (New York, Illinois, and California) chosen based on the highest county-level COVID-19 fatalities as of June 15, 2020. This contextualized studying the pertinent factors in the midst of the first COVID wave in three states that had the highest cumulative county-level COVID-19 fatalities up to June 15. However, the analysis did not adjust for COVID-19 testing capacities for each state. Fatality data was also recorded differently per state (confirmed versus presumed deaths) and may not account for Americans who died of COVID-19 before being tested. Although this is a limitation, this applies to other major national datasets, making our data the best available at the time. County attributed data is restricted to the latest published appropriate year. While AUC and model validation results exhibit room for improvement using tree modeling, these results may be influenced by the limited sample size of the present analysis. Our limited sample size also explains our population density inclusion criteria, as we did not have adequate representation of densely populated counties of less than 1.5 persons or greater than 50,000 persons per square mile.

## Conclusions

To our knowledge, this is the first study to employ a novel decision tree method, which utilized machine-learning techniques to study associations between SDoH and COVID-19 fatalities. The findings support the influence of SDoH on health outcomes, including COVID-19 outcomes, and display the success of decision tree analysis used in this context. Our key finding was that pockets of segregation exist among less densely populated counties and are suggested to predispose those residents to disproportionate COVID-19 outcomes. These areas should be targeted for county-level attention/intervention on multiple levels, including resource planning and allocations. Suggested interventions include increasing educational and employment opportunities as well as community financial resources in these counties identified to be at high risk of COVID-19 fatality. Further research should be directed towards examining the current infrastructures that allow segregation to continue and effective interventions.
